# Environmental exposure as an independent risk factor of chronic bronchitis in northwest Russia

**DOI:** 10.3402/ijch.v72i0.19742

**Published:** 2013-02-22

**Authors:** Pentti Nieminen, Dmitry Panychev, Sergei Lyalyushkin, German Komarov, Alexander Nikanov, Mark Borisenko, Vuokko L. Kinnula, Tuula Toljamo

**Affiliations:** 1 Medical Informatics and Statistics Research Group, University of Oulu, Oulu, Finland; 2 Ministry of Health and Social Development, Murmansk Region, Murmansk, Russia; 3 Monchegorsk City Hospital, Murmansk, Russia; 4 Department of Pulmonary, Murmansk Regional Hospital, Murmansk, Russia; 5 Nord–West Science Centre of Public Health Care, Murmansk, Russia; 6 Department of Medicine, Pulmonary Division, University of Helsinki, Helsinki, Finland; 7 Department of Pulmonary Medicine, Lapland Central Hospital, Rovaniemi, Finland

**Keywords:** sulphur dioxide, pollution, respiratory symptoms, Murmansk, mining

## Abstract

**Background:**

In some parts of the northwest Russia, Murmansk region, high exposures to heavy mining and refining industrial air pollution, especially sulphur dioxide, have been documented.

**Objective:**

Our aim was to evaluate whether living in the mining area would be an independent risk factor of the respiratory symptoms.

**Design:**

A cross-sectional survey of 200 Murmansk region adult citizens was performed. The main outcome variable was prolonged cough with sputum production that fulfilled the criteria of chronic bronchitis.

**Results:**

Of the 200 participants, 53 (26.5%) stated that they had experienced chronic cough with phlegm during the last 2 years. The prevalence was higher among those subjects living in the mining area with its high pollution compared to those living outside this region (35% vs. 18%). Multivariable regression model confirmed that the risk for the chronic cough with sputum production was elevated in a statistical significant manner in the mining and refining area (adjusted OR 2.16, 95% CI 1.07–4.35) after adjustment for smoking status, age and sex.

**Conclusions:**

The increased level of sulphur dioxide emitted during nickel mining and refining may explain these adverse health effects. This information is important for medical authorities when they make recommendations and issue guidelines regarding the relationship between environmental pollution and health outcomes.

Ever more attention is being paid to global pollution and the development of chronic obstructive pulmonary disease (COPD). There is bad industrial pollution in the northwestern part of Russia, mainly in the Kola Peninsula (Murmansk region; Fig. 1), a neighbouring Arctic area that is very close to non-polluted Northern European countries such as Norway and Finland (1,2). Emissions of sulphur dioxide and metals by the nickel industry in the Kola Peninsula seem to be the most important causes of environmental damage (3). Possible adverse health effects have generated considerable concern about public health in Russia, Norway and Finland. Earlier health outcome studies have investigated reproductive and developmental health effects in pregnant women and their new-born babies in the Murmansk region of northwest Russia (1,2,4–6), but no studies have evaluated the symptoms of chronic bronchitis in association with pollution in that particular area.

**Fig. 1:**
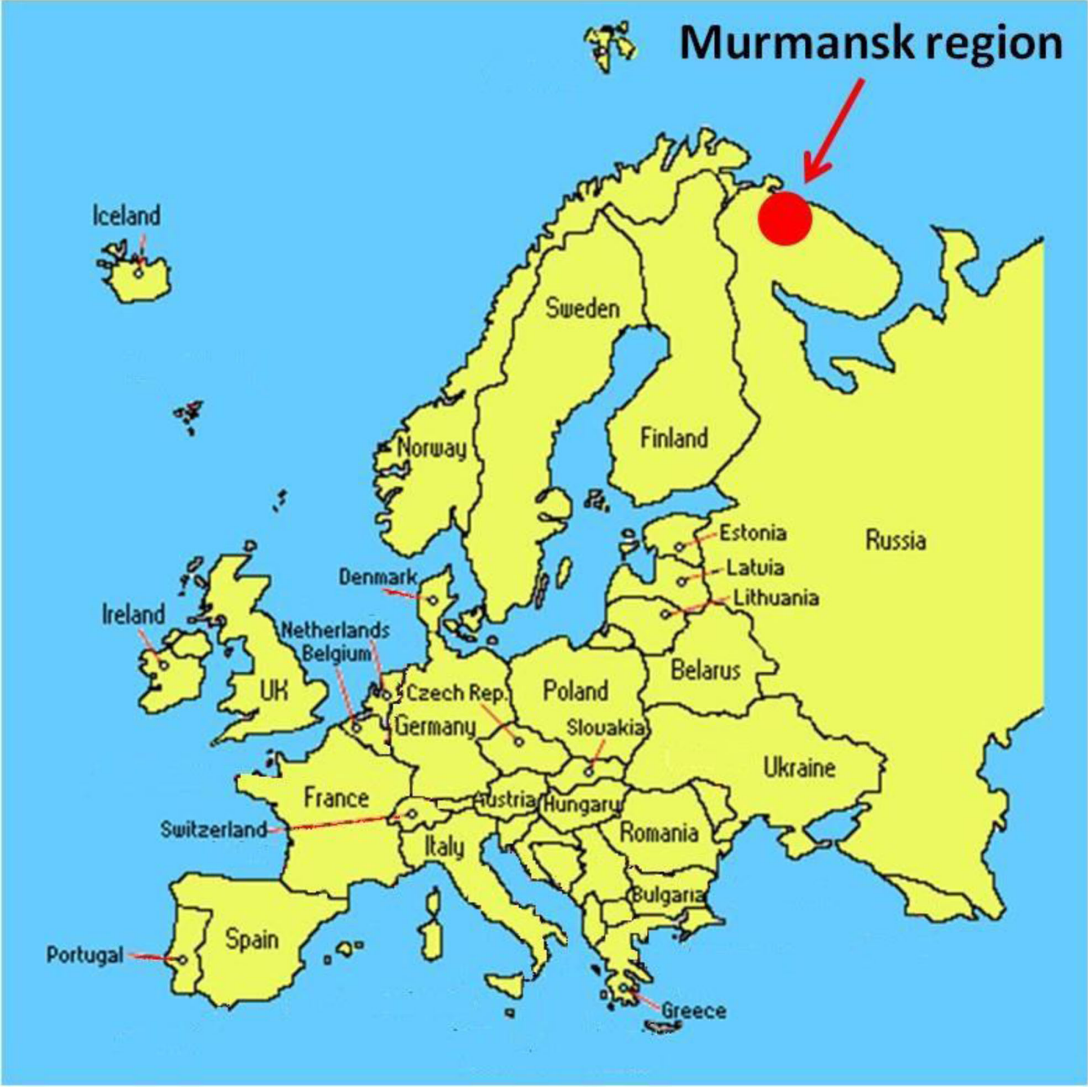
Murmansk region in the northwest Russia.

COPD is strongly related to smoking (7). An international cohort study revealed that the presence of chronic cough/phlegm identifies a subgroup of subjects with a high risk of developing COPD, independently of smoking habits (8). Earlier consistent evidence emphasized that even non-smokers may develop chronic airflow obstruction (9,10). Inhalational exposures such as occupational dusts and chemicals are harmful environmental contaminants (11). The Murmansk region includes areas where its inhabitants are exposed to contaminants from mining industries. The prevalence of smoking (12) and COPD (13) is high in Russia, though the simple statistics probably do not reflect the scale of the problem in this region. There is an urgent need to investigate the persistence of chronic cough/phlegm symptoms by using specific questions about their predictors, onset and duration.

The aims of this analysis were to assess (1) the prevalence of symptoms of chronic bronchitis (or chronic cough/phlegm) among adult subjects who visit a GP for primary care and (2) to test whether a dwelling place and working close to mining industries would be an independent predictor of chronic cough with sputum production.

## Methods

### Background about the region

The study subjects consisted of Murmansk region adult citizens in 2010. The Murmansk Oblast is a federal region of Russia, located in the northwestern part of Russia, mainly in the Kola Peninsula (see Fig. 1). Its administrative centre is the city of Murmansk, with over 300,000 inhabitants. The Murmansk region is a part of the larger Lapland region that spans over 4 countries (Russia, Finland, Norway and Sweden). The Murmansk region is very rich in natural resources. The main industries of the region lie in the sphere of raw material extraction and basic processing. In the southern part of the Murmansk region, the following mineral-based industries are being conducted: iron mining and refining, uranium and thorium recovery, nickel mining and refining (Monchegorsk), apatite recovery, nuclear power generation and aluminium refining (1,14).

The largest industrial plant is located at Monchegorsk, where the operation consists of ore smelting and converting as well as refining the product into pure nickel. Although there are local ore supplies in the Kola Peninsula, sulphur-rich ore is also shipped in from other parts of Russia. Ore roasting, smelting and conversion release large quantities of sulphur dioxide into the environment. The hourly mean reference limit of 350 µg/m^3^ (recommended by European Union) is believed to be exceeded often (3,15). During the last 10 years, there has been active work in modernizing the production of nickel in the Kola Peninsula. As a result of these improvements the sulphur levels have reduced. In conjunction with the metal pollution, sulphur dioxide is still the major environmental contaminant in this area.

The geographic sites in our study were Monchegorsk and Murmansk. Murmansk is the largest city in the Kola Peninsula, with no major metal refining plants. It was included here because it represents an urban community.

### Participants

A total of 200 adult patients, who visited a primary care GP for any medical reason, were enrolled. Venues included one polyclinic located in Murmansk city and one hospital outpatient clinic in the Monchegorsk mining area 150 km outside Murmansk. The study subjects included consecutively enrolled patients and were not actively selected. All participants were interviewed by one physician in Murmansk city and another in Monchegorsk during March and May in 2010. The estimate of sample size was made on the basis of the knowledge that approximately 10–11% of the population in Lapland suffers from pulmonary symptoms (16). We postulated that an increase in symptoms in up to 30% in adults living in this large mining area would be clinically important. An increase of this size with a two-tailed *p*-value of 0.05 and a power of 0.80 would require a sample size of 100 for each dwelling place group. The study design included stratification by gender. The final sample included 50 males and 50 females from 2 clinics. None of the patients visiting a GP refused to participate in the study or were excluded.

### Questionnaire

The questionnaire was developed from a Finnish prospective study for early detection of COPD questionnaire (17) and from the OLIN study questionnaires, which have been used in several Nordic epidemiologic studies (1,11,16,18–20). During the visit to the GP, the doctor interviewed the patient and recorded basic characteristics (age, sex, occupation, exposure to dust). Symptoms (chronic cough with sputum production), smoking status, Smoking Heaviness Index (SHI) and motivation to quit smoking were assessed in the questionnaire for each participant.

Lung functions measured by spirometry were not available for all participants. In the outpatient clinics located in the Murmansk area, the primary GPs book scheduled appointments for spirometry, if necessary.

The Ethical Committee in Ministry of Health and Social Development, Murmansk Region, Russia approved the study. The researchers disclosed information to the participants about the study before they were asked to complete the questionnaire form during the visit. This information included the nature of the survey, assurance that participation in the survey was voluntary, assurance that participation would not cause any harm or delay in their treatment as well as a guarantee of protection of confidentiality.

### Variables

The main outcome variable was assessed by a question: “Have you brought up phlegm when coughing on most days or nights for at least 3 months during the last 2 years?”.

Smoking status (never a smoker, ex-smoker and current smoker) was used as a covariate since smoking is a well-known risk factor of COPD and other respiratory diseases (7). Sex and age (18–30, 31–49, 50–59 and 60–89 years) were used to adjust for their effect on population health.

### Statistical Methods

The data analyses were performed using IBM SPSS Statistics 20.0 software. Cross-tabulation was used to estimate the distributions of chronic cough with sputum according to dwelling place, smoking status, sex and age. In the cross-tabulation, statistical significances of the associations between the chronic cough with sputum and explanatory variables were evaluated with the chi-square test. Multivariable analysis was performed using a dichotomous logistic regression model to investigate the independent effect of dwelling place on the risk of chronic cough with sputum production when smoking status, sex and age were adjusted. The logistic model was reported using crude and adjusted odd ratios (OR) and their 95% confidence intervals (CIs).

## Results

Of the 200 participants, 53 (26.5%) informed us that they had suffered chronic cough with sputum production during the last 2 years (Table I). The symptom was more common in Monchegorsk (35.0% with 95% CI 26.4–44.7) than in Murmansk (18.0% with 95% CI 11.7–26.7) (*p*-value of chi-square test =0.006). The proportion of current smokers was 31.5%. The chronic productive cough was elevated among current smokers as compared to non-smokers. The prevalence of this symptom increased with age (Table I).

**Table I:** Distribution of chronic cough with sputum production by dwelling place, smoking status, age and sex

	Chronic cough with sputum production			
		
Variable	No *n* (%)	Yes *n* (%)	Total	*p-*Value of chi-square test
Dwelling place				0.006
Murmansk	82 (82.0)	18 (18.0)	100 (100)	
Monchegorsk	65 (65.0)	35 (35.0)	100 (100)	
Smoking status				0.023
Never a smoker	79 (81.4)	18 (18.6)	97 (100)	
Ex-smoker	29 (72.5)	11 (27.5)	40 (100)	
Current smoker	39 (61.9)	24 (38.1)	63 (100)	
Sex				0.078
Female	68 (68.0)	32 (32.0)	100 (100)	
Male	79 (79.0)	21 (21.0)	100 (100)	
Age				0.414
10–30 years	36 (81.8)	8 (18.2)	44 (100)	
31–49 years	47 (74.6)	16 (25.4)	63 (100)	
50–59 years	34 (70.8)	14 (29.2)	48 (100)	
60–89 years	30 (66.7)	15 (33.3)	45 (100)	
All	147 (73.5)	53 (26.5)	200	

**Fig. 2:**
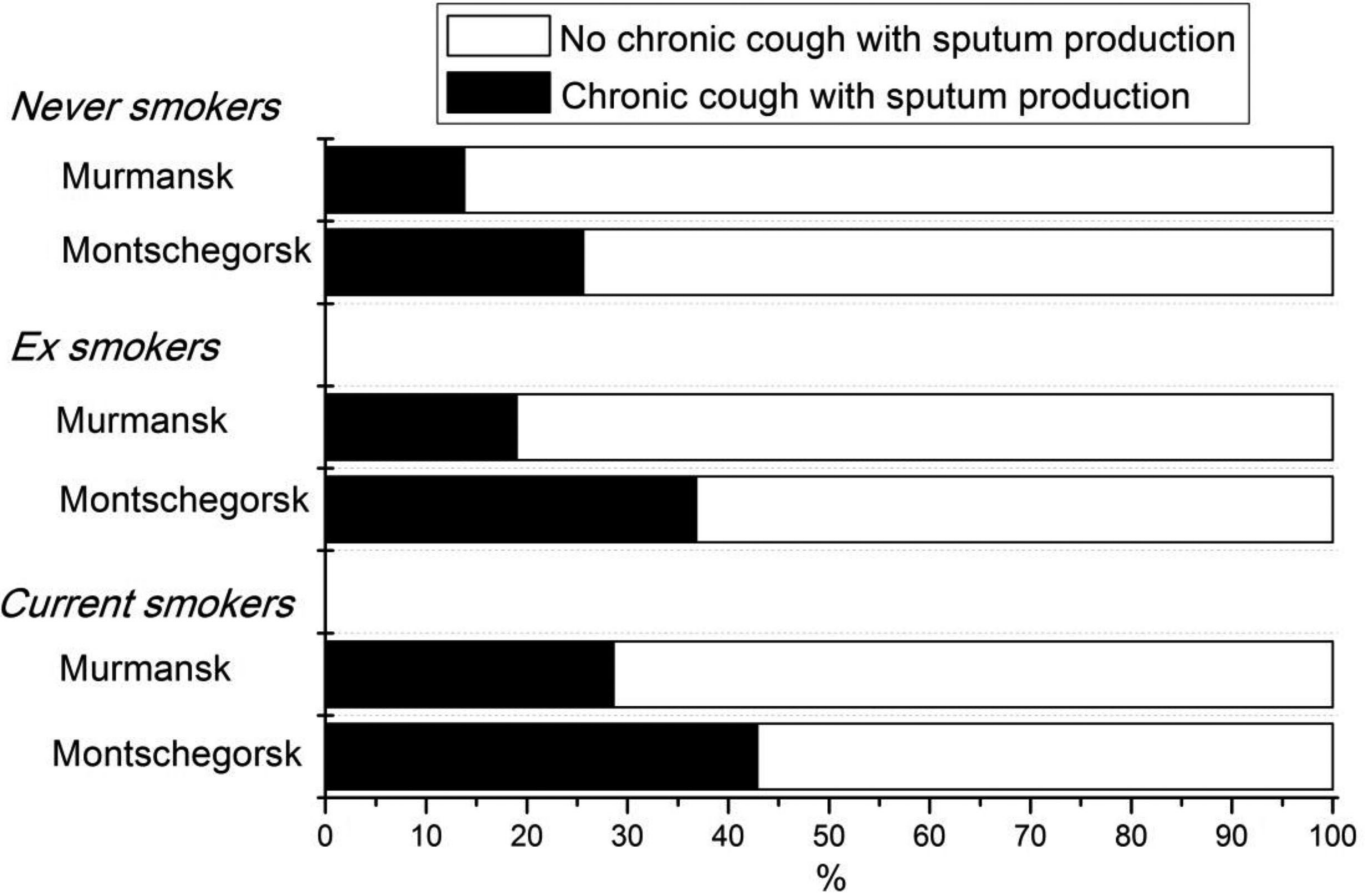
The percentage distribution of sputum production according to smoking status and dwelling place.

Figure 2 presents the subgroup analysis of prolonged cough and sputum production distribution subdivided according to dwelling place in the 3 different smoking status groups. The association between the symptom variable and the dwelling place was present in all smoking groups.

Logistic regression analysis was utilized to study the independent effect of region (dwelling place) on the respiratory symptoms after adjustment for smoking status, age and sex. Table II presents the unadjusted and adjusted odd ratios with their 95% CI. The adjusted model revealed that the risk for the chronic cough with sputum production was higher in Monchegorsk as compared to Murmansk (adjusted OR 2.16, 95% CI 1.07–4.35) after adjustment for smoking status, age and sex. There was no interaction between dwelling place and smoking habits on the risk of chronic cough with sputum production.

**Table II:** Important variables, unadjusted and adjusted odds ratios with 95% confidence interval estimates predicting the probability of chronic cough with sputum production using logistic regression analysis

Variable	Unadjusted OR (95% CI)	Adjusted OR (95% CI)
Dwelling place		
Murmansk (reference)	1	1
Monchegorsk	2.45 (1.27–4.72)	2.16 (1.07–4.35)
Smoking status		
Never smoker (reference)	1	1
Ex-smoker	1.67 (0.70–3.94)	1.89 (0.70–5.07)
Current smoker	2.70 (1.31–5.56)	3.39 (1.40–8.21)
Sex		
Female (reference)	1	1
Male	1.77 (0.93–3.35)	1.18 (0.56–2.47)
Age		
10–30 years (reference)	1	1
31–49 years	1.53 (0.59–3.97)	1.67 (0.62–4.54)
50–59 years	1.85 (0.69–4.97)	2.69 (0.92–7.90)
60–89 years	2.25 (0.84–6.03)	4.47 (1.45–13.83)

Current smoking also increased the risk of chronic productive cough (OR 3.39, 95% CI 1.40–8.21) (Table II). In the Monchegorsk area, smokers were more often males (71.4% vs. 47.6% in Murmansk). Among current smokers, the proportion of older adults was lower than in the total sample, but there was no difference between dwelling places. Most (57.1%) of the current smokers were not heavily nicotine dependent according to their HIS value. Only 42.9% of current smokers displayed a high motivation to quit smoking.

## Discussion

We studied the prevalence of chronic cough with sputum production in relation to the dwelling place and environmental contaminants in the northwestern part of Russia. The main findings of our investigation were as follows: (1) the prevalence of chronic cough with sputum production was rather high in both the Murmansk city where there is no major metal producing industries (18%) and in Monchegorsk with its wide nickel refining operations (35%), and (2) inhabitants living in the area with high environmental pollution due to mining are at an increased risk of suffering chronic cough and sputum production (chronic bronchitis), independently of their smoking habits.

COPD is a major economic and social burden for the individual patient and society as a whole. This study revealed that self-reported chronic symptoms were common in northwestern Russia, 28.5% of participants reported the presence of cough and 26.5% sputum production on most days. The prevalence of chronic cough was much higher than the corresponding value reported in a study from northern Finland and Sweden (16). Lindström et al. (16) found that the prevalence of chronic productive cough among adults was 11.0% in Lapland, Finland, and 7.4% in Norrbotten, Sweden. These 2 areas have similar geographic conditions than Kola Peninsula in Russia. In a cohort of young Finnish military draftees from Lapland, northern Finland, the prevalence of chronic cough with sputum production was 12% in non-smokers, 26.9% in occasional smokers and 40.7% in regular smokers (21). Another study from northern Finland found that after detailed questionnaires, a total of 186/544 (34.2%) of adult current smokers reported both cough and sputum production even though they considered themselves as being symptom-free and healthy at the very beginning of the study (17). Thus, when compared to these studies from the northern neighbouring areas, the symptoms of chronic cough and sputum production in our study are not exceptionally high.

There was a remarkable relationship between dwelling place and respiratory production. Patients living in the polluted area reported more often chronic bronchitis, especially sputum production. This association was independent of their smoking status. The main difference between the 2 dwelling places was the large-scale nickel mining and refining undertaken in Monchegorsk. The presence of chronic cough with sputum production can be high independently of smoking habits (7). Environmental contaminants such as air pollution are known to be risk factors of respiratory diseases (22,23). In particular, the increased levels of sulphur dioxide have been reported to increase hospital admissions for chronic bronchitis and the frequency of wheeze (22,24–26).

Nickel mining and refining release sulphur dioxide (27). Sulphur dioxide is a major air pollutant and it has a significant impact on human health. Sulphur dioxide is a colourless gas that is very soluble in water. Since it is very soluble, sulphur dioxide is readily absorbed in the upper respiratory tract and its effects are enhanced if penetration into lower regions is increased (through mouth rather than nose breathing and through exercise that elevates the amount and depth of inhalation). Inhaling sulphur dioxide irritates both the upper and lower airways. Its typical symptoms include cough, sputum production and shortness of breath. Cold weather can increase these symptoms, at least in those individuals with smoking related airway disease in northern areas (28). A study carried out in the Norwegian–Russian border investigated the effects of daily variations in sulphur dioxide on the lung function levels of people living on both sides of the border (15). None of the study populations exhibited a detectable reduction in lung function when the sulphur dioxide increased above the reference level. However, our study now indicates that the presence of chronic cough and sputum production was higher in the area where there are high sulphur dioxide levels present in the environment.

A major limitation of this study was that the study subjects were not drawn from the general population living in the study areas. For practical reasons, the study subjects included only local adult residents who visited a primary care GP for any medical reason. The visit made it possible for the GP to interview the patient, record basic characteristics (age, sex, smoking status occupation, exposure to dust) and assess the symptoms. Thus, the obtained prevalence of chronic bronchitis in Murmansk city or in Monchegorsk mining area may be affected by a selection bias. However, for public health authorities, this estimate of disease prevalence is valuable.

Another limitation of this study was that no accurate assessment of air pollution exposure was available and that the exposure was a pollutant mixture, though the later situation is the case in most real-life scenarios. The available information from the Monchegorsk area confirmed that roasting, smelting, and conversion of metal ores release huge quantities of sulphur dioxide, which, together with the release of metals, has caused an ecological catastrophe in the areas immediately adjacent to the mining operations (1,3). Sulphur dioxide seems to be the most important cause of both environmental and adverse health effects in Monchegorsk. The area outside the pollution zone (20–30 km from Monchegorsk) can be considered as pristine and a very unspoiled area.

In global terms, tobacco smoke remains the most important cause of respiratory symptoms and COPD (7,29) and elimination of this risk factor would represent an important step toward reducing the COPD incidence. However, other risk factors should also be taken into account in the prevention and control of COPD. Exposure to occupational dust, chemicals and fumes are important factors for many patients with COPD (29–32). Exposure to indoor air pollution from sulphur-containing biomass fuels, which are used for cooking and heating in poorly ventilated homes, might be a potential risk factor for COPD in non-smokers – especially in women living in developing countries (33–35). Our results also suggest that outdoor air pollution, e.g. high sulphur dioxide levels, is linked to chronic cough and sputum production in areas close to the European Union and that those in turn are important in both the development and progression of COPD.

It is important to increase our awareness of the relationships between environmental pollution and health outcomes. This information is important for medical authorities when they make recommendations and issue guidelines for the population. The general public, especially patients with upper or lower respiratory symptoms, are now aware from media reports that adverse respiratory effects can be attributed to environmental pollution related to industry (22). However, GPs or respiratory physicians need to have accurate and up-to-date knowledge of the potential health effects of air pollution and how they might affect their patients in order to advise them appropriately.
